# Comparative Study of Monoexponential, Intravoxel Incoherent Motion, Kurtosis, and IVIM-Kurtosis Models for the Diagnosis and Aggressiveness Assessment of Prostate Cancer

**DOI:** 10.3389/fonc.2020.01763

**Published:** 2020-09-11

**Authors:** Ying Liu, Xuan Wang, Yadong Cui, Yuwei Jiang, Lu Yu, Ming Liu, Wei Zhang, Kaining Shi, Jintao Zhang, Chen Zhang, Chunmei Li, Min Chen

**Affiliations:** ^1^Department of Radiology, Beijing Hospital, National Center of Gerontology, Institute of Geriatric Medicine, Chinese Academy of Medical Sciences, Beijing, China; ^2^Department of Radiology, Civil Aviation General Hospital, Civil Aviation Clinical Medical College of Peking University, Beijing, China; ^3^Department of Urology, Beijing Hospital, National Center of Gerontology, Institute of Geriatric Medicine, Chinese Academy of Medical Sciences, Beijing, China; ^4^Department of Pathology, Beijing Hospital, National Center of Gerontology, Institute of Geriatric Medicine, Chinese Academy of Medical Sciences, Beijing, China; ^5^Philips Healthcare, Beijing, China

**Keywords:** monoexponential model, intravoxel incoherent motion model, kurtosis model, IVIM–kurtosis model, prostate cancer, prostatitis, benign prostatic hyperplasia

## Abstract

**Objective:** This study aimed to compare the potential of monoexponential model (MEM), intravoxel incoherent motion (IVIM) model, kurtosis model, and IVIM–kurtosis model in the diagnosis and aggressiveness assessment of prostate cancer (PCa).

**Materials and Methods:** Thirty-six patients were recruited. Diffusion-weighted images were acquired on a 3.0-T magnetic resonance imaging (MRI) system using 0 *b* values up to 2,000 s/mm^2^ and analyzed using four models: MEM (ADC_MEM_), IVIM (*D*_IVIM_, D*_IVIM_, *f*_IVIM_), kurtosis (*D*_kurtosis_, *K*_kurtosis_), and IVIM–kurtosis (*D*_IVIM−kurtosis_, D^*^_IVIM−kurtosis_, *f*_IVIM−kurtosis_, *D*_IVIM−kurtosis_) models. The values of these parameters were calculated and compared between PCa, benign prostatic hyperplasia (BPH), and prostatitis. Correlations between these parameters and the Gleason score (GS) of PCa were evaluated using the Pearson test.

**Results:** Forty-five lesions were studied, including 18 PCa, 12 prostatitis, and 15 BPH lesions. The ADC_MEM_, *D*_IVIM_, *f*_IVIM_, *D*_kurtosis_, and *D*_IVIM−kurtosis_ values were significantly lower and *K*_kurtosis_ and *K*_IVIM−kurtosis_ values were significantly higher in PCa compared with prostatitis and BPH. The area under the curve (AUC) of ADC_MEM_ showed significantly higher values than that of *f*_IVIM_ and *K*_IVIM−kurtosis_, but no statistical differences were found between the other parameters. The D^*^_IVIM−kurtosis_ value correlated negatively and *f*_IVIM−kurtosis_ and *K*_IVIM−kurtosis_ values correlated positively with the GS.

**Conclusion:** The MEM, IVIM, kurtosis, and IVIM–kurtosis models were all useful for the diagnosis of PCa, and the diagnostic efficacy seemed to be similar. The IVIM–kurtosis model may be superior to the MEM, IVIM, and kurtosis models in the grading of PCa.

## Introduction

Prostate cancer (PCa) is the second-most frequent cancer and the fifth leading cause of cancer-related mortality in men worldwide (1). It is critical to accurately detect PCa and assess its aggressiveness. The European Society of Urogenital Radiology prostate committee highlights the use of magnetic resonance imaging (MRI) in managing suspected PCa (2).

MRI is an excellent technique for detecting and assessing the aggressiveness of PCa, because it can provide both anatomic and functional information. Among various MRI techniques available, diffusion-weighted imaging (DWI) shows great potential to be applied as a clinical marker of tumor diagnosis and aggressiveness assessment (3–5). The apparent diffusion coefficient (ADC), a parameter derived from DWI assuming a monoexponential model (MEM), is usually lower in PCa compared with benign prostate tissues, which is attributed to the increased cellularity of proliferating PCa. Moreover, several previous studies have shown statistically significant correlations between the ADC and the Gleason score (GS) of PCa (6, 7). However, overlaps exist between quantitative ADC values derived from PCa with a higher and a lower GS, as well as between those derived from benign prostatic tissues (8). This may be due to a drawback of the MEM, which assumes a Gaussian distribution of the water thermal motion. Actually, in biological tissues, water diffusion is restricted by the presence of barriers and compartments, leading to a non-Gaussian distribution (9, 10). Therefore, non-Gaussian diffusion models have been introduced for the study of DWI, such as intravoxel incoherent motion (IVIM) and diffusion kurtosis imaging (DKI). IVIM was originally described by Le Bihan (11) and Le Bihan et al. (12), while DKI was first described by Jensen et al. in 2005 (13). Several studies have explored the values of IVIM and DKI for the diagnosis and aggressiveness assessment of PCa. The results have proved that IVIM (14–19) and DKI (20–24) may contribute to the detection and aggressiveness assessment of PCa. However, the results of IVIM and DKI for assessing the aggressiveness of PCa were various; several studies showed that IVIM (16–18) and DKI (22–24) were feasible to stratify the pathological grade of PCa, but a few studies found negative results of IVIM (15) and DKI (20) parameters in predicting the GS.

The IVIM–kurtosis model, taking into account altogether IVIM and non-Gaussian diffusion effects on the diffusion-weighted signal, can provide more parameters compared with the IVIM and DKI models. However, no study has evaluated the utility of the IVIM–kurtosis model in the detection and staging of PCa.

No study has compared the efficiency of the MEM, IVIM, kurtosis, and IVIM–kurtosis models in the diagnosis and aggressiveness assessment of PCa in the same series of patients. This study aimed to quantitatively compare the utility of parameters obtained from the MEM, IVIM, kurtosis, and IVIM–kurtosis models on the differential diagnosis and aggressiveness assessment of PCa, and taking in-bore transrectal MRI-guided biopsy as a pathological reference.

## Materials and Methods

### Patient Population

**Figure 1:**
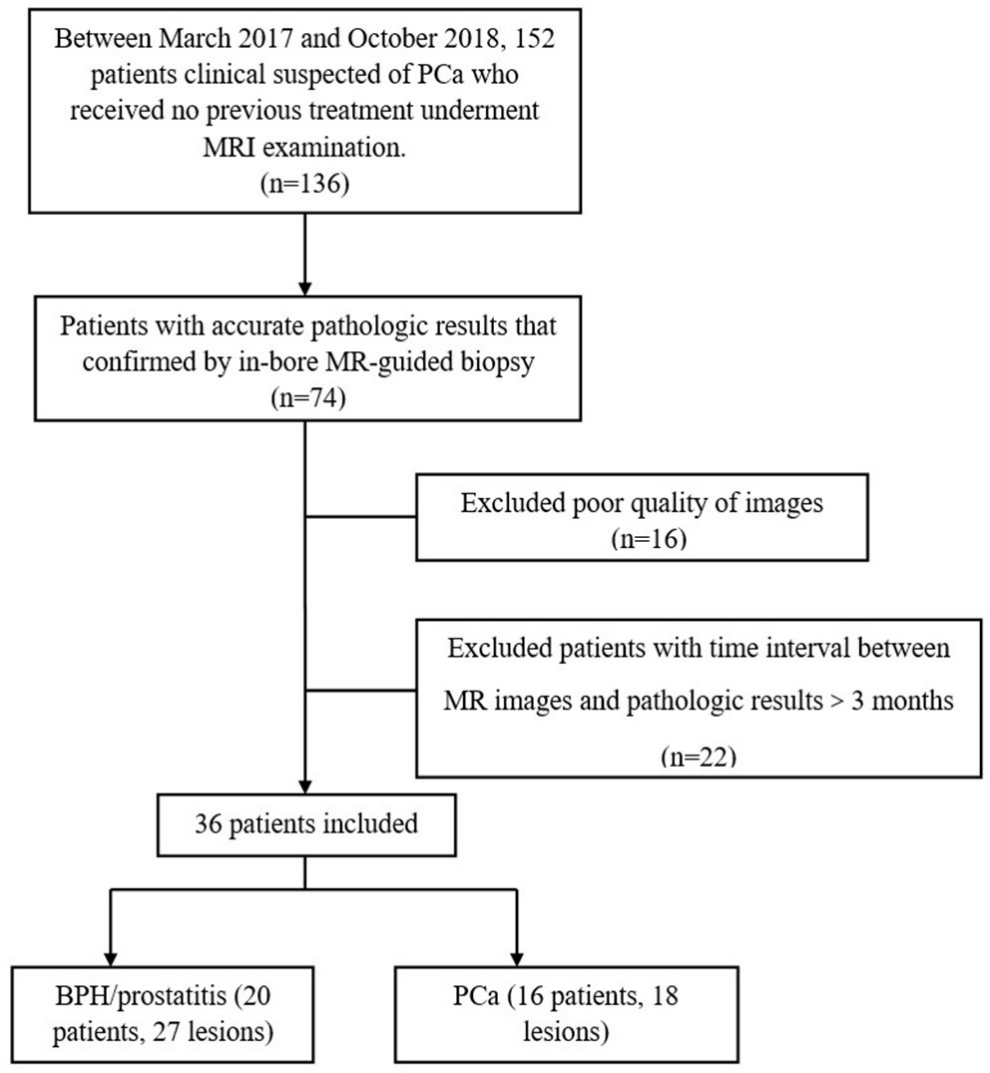
General clinical and pathological information of the patients.

This prospective study was approved by the local institutional review board, and written informed consent was obtained from each patient before the study. Between March 2017 and October 2018, this study included 152 patients with clinical suspicion of PCa. The inclusion criteria were as follows: (a) Routine MRI and multi-*b* value DWI images of the prostate were acquired before the in-bore transrectal MRI-guided puncture. (b) MRI was performed in patients prior to any treatment of PCa. The exclusion criteria were as follows: (a) images with poor quality, which were regarded as inadequate for the following analysis; (b) patients without pathologic results that proven by subsequent in-bore transrectal MRI-guided prostate biopsy; and (c) time interval between the MRI examination and MRI-guided prostrate biopsy ≥3 months. Figure 1 shows the flow diagram of the recruitment process. Finally, 36 patients were included in this study.

### Magnetic Resonance Imaging

All examinations were performed on a 3.0-T MRI scanner (Achieva, Philips Medical Systems, Best, the Netherlands) with an eight-channel cardiac coil. The scan protocol included axial, coronal, and sagittal T2-weighted image turbo spin echo (TSE); axial T2WI TSE spectrally selective attenuated inversion recovery (SPAIR); and axial T1-weighted image TSE, axial DWI, and multi-*b* value DWI. The multi-*b* value DWI was acquired using the following imaging parameters: repetition time (TR) = 5,131 ms, echo time (TE) = 63 ms, thickness/gap = 4/1 mm, field of view (FOV) = 220 × 200 mm SENSE factor = 2, matrix sizes = 88 × 79, gradient overplus = yes, gradient mode = maximum; and *b* values = 0, 20, 50, 100, 200, 500, 1,000, 1,500, and 2,000 s/mm^2^. The diffusion gradient duration is 28.2 and 40.5 ms with ramp time. The acquisition time for this sequence was 7 min and 57 s.

### In-bore MRI-Guided Prostate Biopsy

The targeted biopsy of suspicious lesions was conducted on the MRI scanner, using an MRI-compatible biopsy device (*Invivo*, Schwerin, Germany) and an MRI-guided biopsy system (DynaCAD version 2.1.8). The suspicious lesions were identified by two radiologists (MC and CML, with 20 and 10 years of MRI experience, respectively) according to the Prostate Imaging-Reporting and Data System, version 2. The lesions were hypointense on T2WI, hyperintense on DWI, and hypointense on ADC. In the process of puncture biopsy, one radiologist (CL) performed the lesion localization, another (JY or XD) performed the interventional biopsy, and a radiology technologist (JZ) performed the scanning operation. Finally, 36 patients were included in this study: 29 patients had 1 lesion, 5 had 2 lesions, and 2 had 3 lesions. Ultimately, 45 lesions were investigated, including 18 PCa, 12 prostatitis, and 15 BPH.

### Image Data Analysis

Signal-to-noise ratio (SNR) of DWI images with b-value of 2,000 s/mm^2^ is calculated using the following equation:

$$\begin{array}{l} SNR=\frac{avg\left(S_{b=2000}\right)}{\sqrt{avg\left(background\right)}} \end{array}$$

Sb = 2,000 is the signal intensity (SI) of 2 ROIs drawn on peripheral and transitional zone, while background is the SI of 4 ROIs drawn on corners of images.

The multi-*b* value DWI images were processed using the Matlab R2015b. The regions of interest (ROIs) were drawn manually on the puncture lesions on parametric maps, and the ROIs were identified by the consensus of two experienced radiologists (YL and CML, with 3 and 10 years of MRI experience, respectively). The Akaike Information Criteria (AIC) and the parameters (*D*_IVIM_, D*_IVIM_, *f*_IVIM_, *D*_kurtosis_, *K*_kurtosis_, *D*_IVIM−kurtosis_, D*_IVIM−kurtosis_, *f*_IVIM−kurtosis_, and *K*_IVIM−kurtosis_) of IVIM, Kurtosis, and IVIM-kurtosis models were measured at the same time in the post-processing step.

ADC_MEM_ was derived from the MEM, using 50 and 1,500 s/mm^2^ *b* values. *D*_IVIM_, D*_IVIM_, and *f*_IVIM_ were derived from the IVIM model; *D*_kurtosis_ and *K*_kurtosis_ were derived from the kurtosis model; *D*_IVIM−kurtosis_, D*_IVIM−kurtosis_, *f*_IVIM−kurtosis_, and *K*_IVIM−kurtosis_ were derived from the IVIM–kurtosis model. The same ROI was used for these four models.

ADC_MEM_ was calculated by the MEM using the following equation (25):

$$\begin{array}{l} S\left(b\right)/S0=\text{ exp }\left(-b\times ADC\right) \end{array}$$

where *S*(*b*) is the mean signal intensity with diffusion gradient *b, S*0 is the mean signal intensity without diffusion gradient, and *b* is the *b* value (50 and 1,500 s/mm^2^).

The IVIM model and its parameters were fitted according to the following bi-exponential equation (26):

$$\begin{array}{l} S\left(b\right)/S0=f{e^{-bD}}^{*}+{\left(\text{1}-f\right)}^{-bD} \end{array}$$

where *S*(*b*) is the mean signal intensity, *S*0 is the signal reference, *b* represents the *b* value (0, 20, 50, 100, 200, 500, 1,000, 1,500, and 2,000 s/mm^2^), and *f* is the perfusion fraction. D^*^ is the perfusion-related diffusion coefficient, and *D* represents the diffusion of the non-perfusing fraction.

The kurtosis model was expressed by the following equation (13):

$$\begin{array}{l} S\left(b\right)=S\left(0\right)\text{ exp }\left(-bD+b^{6}\cdot D^{6}\cdot K/6\right) \end{array}$$

where *S*(*b*) is the signal intensity at a specified *b* value; *S*0 is the baseline signal intensity at *b* = 0; *b* represents the *b* value (0, 20, 50, 100, 200, 500, 1,000, 1,500, and 2,000 s/mm^2^); *D* represents the non-Gaussian diffusion coefficient; and *K* represents the apparent kurtosis coefficient without unit.

The IVIM–kurtosis model was expressed by the following equation (27):

$$\begin{array}{l} S\left(b\right)=S\left(0\right)\{f_{IVIM}\cdot \text{ exp }\left(-b\cdot D^{*}\right)\\ \;+\left(1-f_{IVIM}\right)\\ \;.\text{ exp }[-b\cdot {\text{ADC}}_{0}\\ \;+{\left(b\cdot {\text{ADC}}_{0}\right)}^{2}\cdot K/6]\} \end{array}$$

where S(0) is the theoretical signal intensity at a b value of 0 s/mm^2^, *f*_IVIM_ represents the (T1-, T2-weighted) volume fraction of incoherently flowing blood in the tissue, b is the b value (0, 20, 50, 100, 200, 500, 1,000, 1,500, and 2,000 s/mm^2^), D^*^ is the pseudo-diffusion coefficient associated with the IVIM effect, ADC_0_ is the virtual ADC obtained when *b* = 0, and *K* represents the kurtosis parameter.

AIC was employed to evaluate the performance of curve fitting (28):

$$\begin{array}{l} AIC=N\text{ln}\left(SSE\right)-N\text{ln}\left(N\right)+2\left(p+1\right) \end{array}$$

where *N* is the number of data points, *SSE* is the sum of squared deviance, and *p* is the number of parameters.

### Statistical Analysis

SPSS 22.0 (IBM Corp, NY, USA) and Medcalc (15.8) were used to perform the statistical analyses. The difference among the AIC of IVIM, Kurtosis and IVIM-kurtosis were calculated by analysis of variance (ANOVA) test. Differences among PCa, BPH, and prostatitis in the parameters ADC_MEM_, *D*_IVIM_, D*_IVIM_, *f*_IVIM_, *D*_kurtosis_, *K*_kurtosis_, *D*_IVIM−kurtosis_, D*_IVIM−kurtosis_, *f*_IVIM−kurtosis_, and *K*_IVIM−kurtosis_ were assessed using the analysis of variance (ANOVA) test.

The diagnostic efficiency of the MEM, IVIM, kurtosis, and IVIM–kurtosis models was calculated using receiver operating characteristics (ROC) curves and the binary logistic regression model. The diagnostic sensitivity and specificity were calculated at a cutoff point that maximized the value of the Youden index. Three logistic regression models were established, with IVIM model (*D*_IVIM_ + *f*_IVIM_), kurtosis model (*D*_kurtosis_ + *K*_kurtosis_), and IVIM–kurtosis model (*D*_IVIM−kurtosis_ + *K*_IVIM−kurtosis_). ROC comparisons among different parameters and models were also performed. The Pearson tests were used to evaluate the correlations between these parameters and GS of PCa. A *P* < 0.05 was considered statistically significant.

## Results

The mean SNRs were 27.29 (range, 19.49–36.59) for peripheral zone and 24.49 (range, 17.55–34.02) for transitional zone on images with b-value of 2,000 s/mm^2^.

Table 1 shows the clinical and pathological characteristics of the patients.

**Table 1:** General clinical and pathological information of the patients.

	**PCa**	**BPH/Prostatitis**	***t***	***P***
Foci number	18	27		
Age (year)	74.38 ± 7.99	68.25 ± 8.39	1.740	0.319
PSA (ng/mL)	15.82 ± 15.60	7.16 ± 4.81	1.520	**0.037**
**ISUP grade/GS**				
1/3+3 = 6	5			
2/3+4 = 7	3			
3/4+3 = 7	4			
4/4+4 = 8	4			
5/4+5 = 9	2			

GS, Gleason Score; ISUP, International Society of Urological Pathology. The bold value means *p* < 0.05.

Table 2 shows the AIC of IVIM, Kurtosis and IVIM-kurtosis models. The AIC of IVIM and IVIM-kurtosis were lower than the AIC of Kurtosis, but no differences were found between the AIC of IVIM and IVIM-kurtosis.

**Table 2:** The AIC of MEM, IVIM, kurtosis, and IVIM-kurtosis models.

	**IVIM**	**Kurtosis**	**IVIM-kurtosis**	**ANOVA**	***p***		
					**(1)**	**(2)**	**(3)**
AIC	−55.09 ± 6.74	−46.22 ± 6.90	−53.18 ± 6.66	**<0.001**	**<0.001**	0.294	**<0.001**

AIC, Akaike Information Criteria.

Three P-values correspond to the results between the AIC of IVIM and Kurtosis, IVIM and IVIM-kurtosis, and Kurtosis and IVIM-kurtosis, respectively. The bold values mean *p* < 0.05.

**Figure 2:**
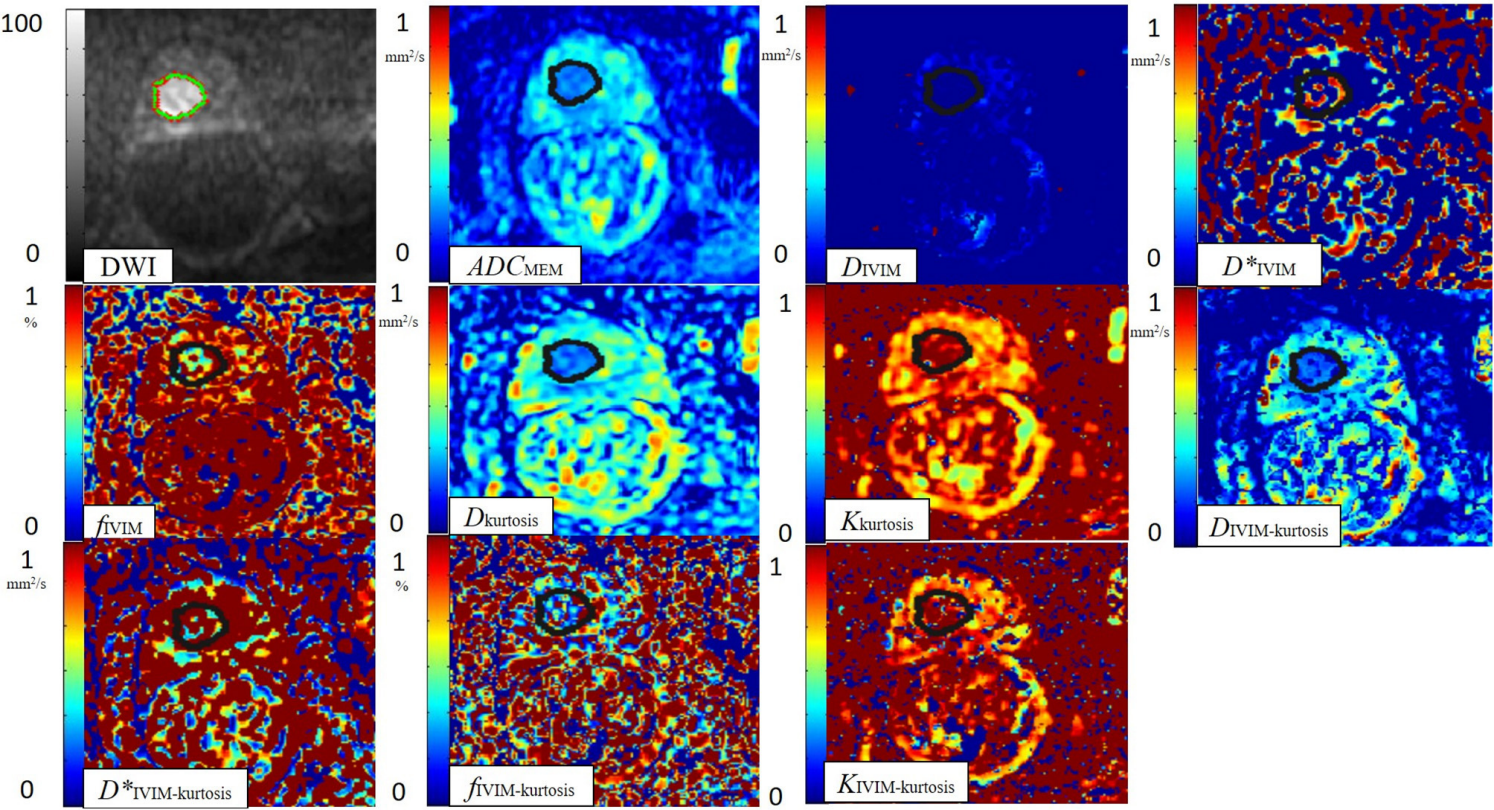
One case with PCa. A 76-year-old man with PCa (pathological GS, 3 + 4). ROI was set in PCa on DWI. ADC_MEM_, *D*_IVIM_, D*_IVIM_, *f*_IVIM_, *D*_kurtosis_, *K*_kurtosis_, D_IVIM−kurtosis_, D*_IVIM−kurtosis_, (j) *f*_IVIM−kurtosis_, and *K*_IVIM−kurtosis_ value maps. The cancer tissue showed different signals notably from normal tissues in the DWI, ADC_MEM_ (0.79 × 10^−3^ mm^2^/s), *D*_IVIM_ (0.39 × 10^−3^ mm^2^/s), *f*_IVIM_ (48.45%), *D*_kurtosis_ (1.24 × 10^−3^ mm^2^/s), *K*_kurtosis_ (0.99), *D*_IVIM−kurtosis_ (0.98 × 10^−3^ mm^2^/s), and *K*_IVIM−kurtosis_ (1.36) value maps. The PCa tissue seemed to be similar to normal tissues on the D*_IVIM_ (5.53 × 10^−3^ mm^2^/s), *f*_IVIM−kurtosis_ (24.09%), and D*_IVIM−kurtosis_ (0.78 mm^2^/s) value maps.

**Figure 3:**
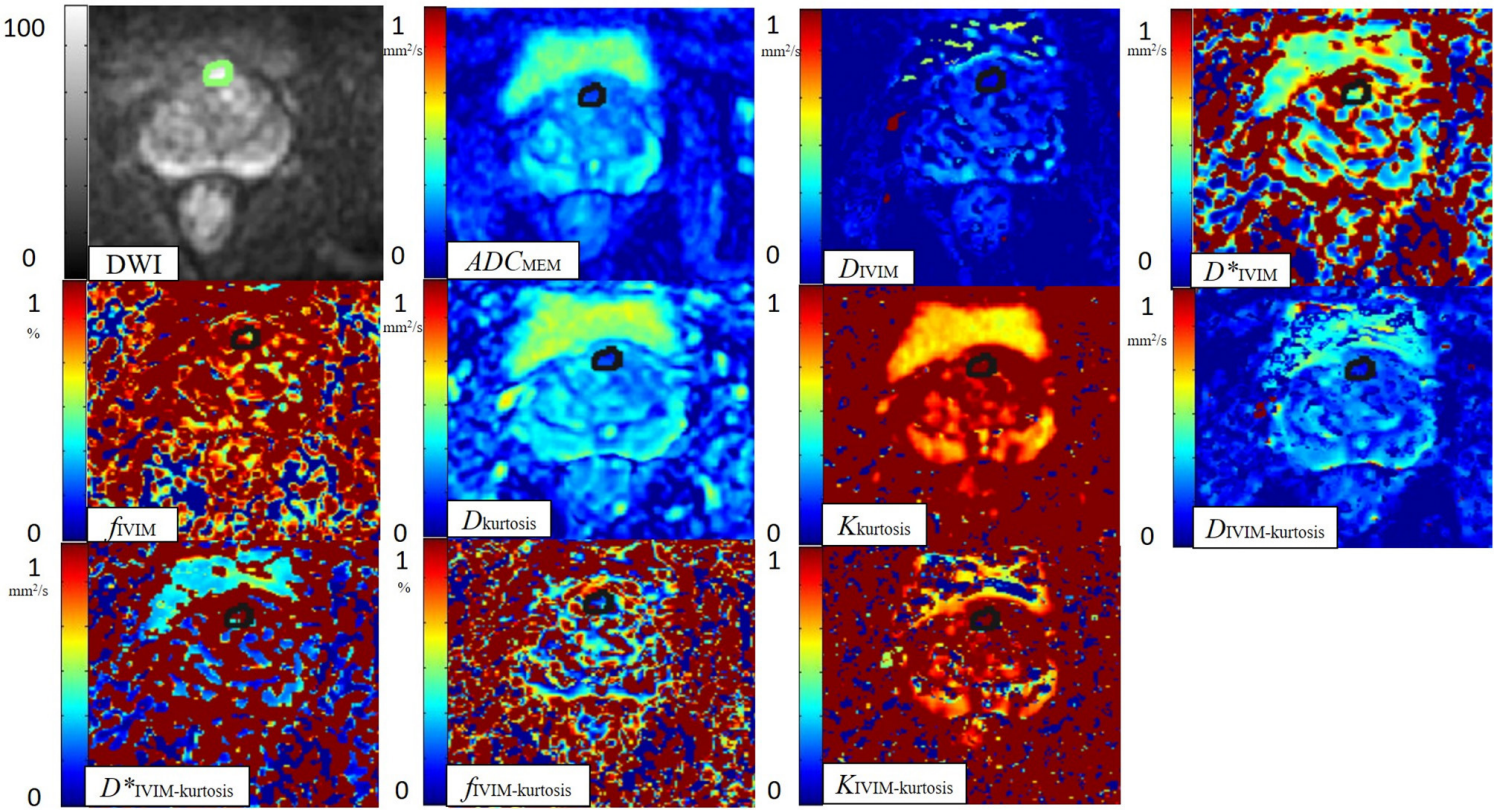
One case with BPH. A 60-year-old man with BPH. ROI was set in BPH on the DWI image. ADC_MEM_, *D*_IVIM_, D*_IVIM_, *f*_IVIM_, *D*_kurtosis_, *K*_kurtosis_, *D*_IVIM−kurtosis_, D*_IVIM−kurtosis_, *f*_IVIM−kurtosis_, and *K*_IVIM−kurtosis_ value maps. The BPH tissue showed different signals notably from normal tissues on the DWI value map. The BPH tissue showed a slightly low signal on the ADC_MEM_ (0.87 × 10^−3^ mm^2^/s), *D*_VIM_ (0.45 × 10^−3^ mm^2^/s), *D*_kurtosis_ (1.34 × 10^−3^ mm^2^/s), and *D*_IVIM−kurtosis_ (1.22 × 10^−3^ mm^2^/s) value maps. The BPH tissue seemed to be similar to normal tissues on the D*_IVIM_ (4.09 × 10^−3^ mm^2^/s), *f*_IVIM_ (46.17%), *K*_kurtosis_ (0.96), D*_IVIM−kurtosis_ (1.65 mm^2^/s), *f*_IVIM−kurtosis_ (15.25%), and *K*_IVIM−kurtosis_ (1.01) value maps.

The ANOVA test results are summarized in Table 3, displaying that ADC_MEM_, *D*_IVIM_, *f*_IVIM_, *D*_kurtosis_, and *D*_IVIM−kurtosis_ values were significantly lower in PCa than in prostatitis and BPH, but no differences were found between prostatitis and BPH. The *K*_kurtosis_ and *K*_IVIM−kurtosis_ values were significantly higher in PCa compared with prostatitis and BPH, but no differences were found between prostatitis and BPH. No significant differences were found among PCa, prostatitis, and BPH for D*_IVIM_, D*_IVIM−kurtosis_, and *f*_IVIM−kurtosis_. One case with PCa and one case with BPH are shown in Figures 2, 3.

**Table 3:** MEM, IVIM, kurtosis, and IVIM-kurtosis model parameter values in PCa, BPH, and prostatitis.

	**PCa**	**BPH**	**Prostatitis**	**ANOVA**	***P***		
	**(*n* = 18)**	**(*n* = 15)**	**(*n* = 12)**		**(1)**	**(2)**	**(3)**
**MEM**							
ADC_MEM_ (10^−3^mm^2^/s)	0.67 ± 0.14	1.01 ± 0.13	1.10 ± 0.28	**<0.001**	**<0.001**	**<0.001**	0.324
**IVIM**							
*D*_IVIM_ (10^−3^mm^2^/s)	0.39 ± 0.13	0.59 ± 0.10	0.63 ± 0.17	**<0.001**	**<0.001**	**<0.001**	0.440
D*_IVIM_ (10^−3^mm^2^/s)	5.36 ± 3.14	6.48 ± 2.64	6.04 ± 2.81	0.603	0.324	0.612	0.754
*f*_IVIM_ (%)	34.67 ± 12.72	47.09 ± 12.47	49.21 ± 13.84	**0.017**	**0.017**	**0.018**	0.731
**Kurtosis**							
*D*_kurtosis_ (10^−3^mm^2^/s)	0.96 ± 0.29	1.55 ± 0.25	1.66 ± 0.43	**<0.001**	**<0.001**	**<0.001**	0.462
*K*_kurtosis_	0.96 ± 0.18	0.75 ± 0.12	0.68 ± 0.09	**<0.001**	**0.001**	**<0.001**	0.394
**IVIM-kurtosis**							
*D*_IVIM−kurtosis_ (10^−3^mm^2^/s)	0.79 ± 0.26	1.28 ± 0.22	1.43 ± 0.50	**<0.001**	**<0.001**	**<0.001**	0.286
D*_IVIM−kurtosis_ (mm^2^/s)	0.77 ± 0.73	1.13 ± 0.61	1.37 ± 0.93	0.175	0.211	0.082	0.498
*f*_IVIM−kurtosis_ (%)	21.79 ± 11.16	22.91 ± 7.67	22.97 ± 11.08	0.946	0.775	0.798	0.989
*K*_IVIM−kurtosis_	1.51 ± 0.86	0.93 ± 0.38	0.62 ± 0.20	**0.008**	**0.025**	**0.004**	0.311

Three P-values correspond to the results between PCa and BPH, PCa and prostatitis, and BPH and prostatitis, respectively. The bold values mean *p* < 0.05.

**Figure 4:**
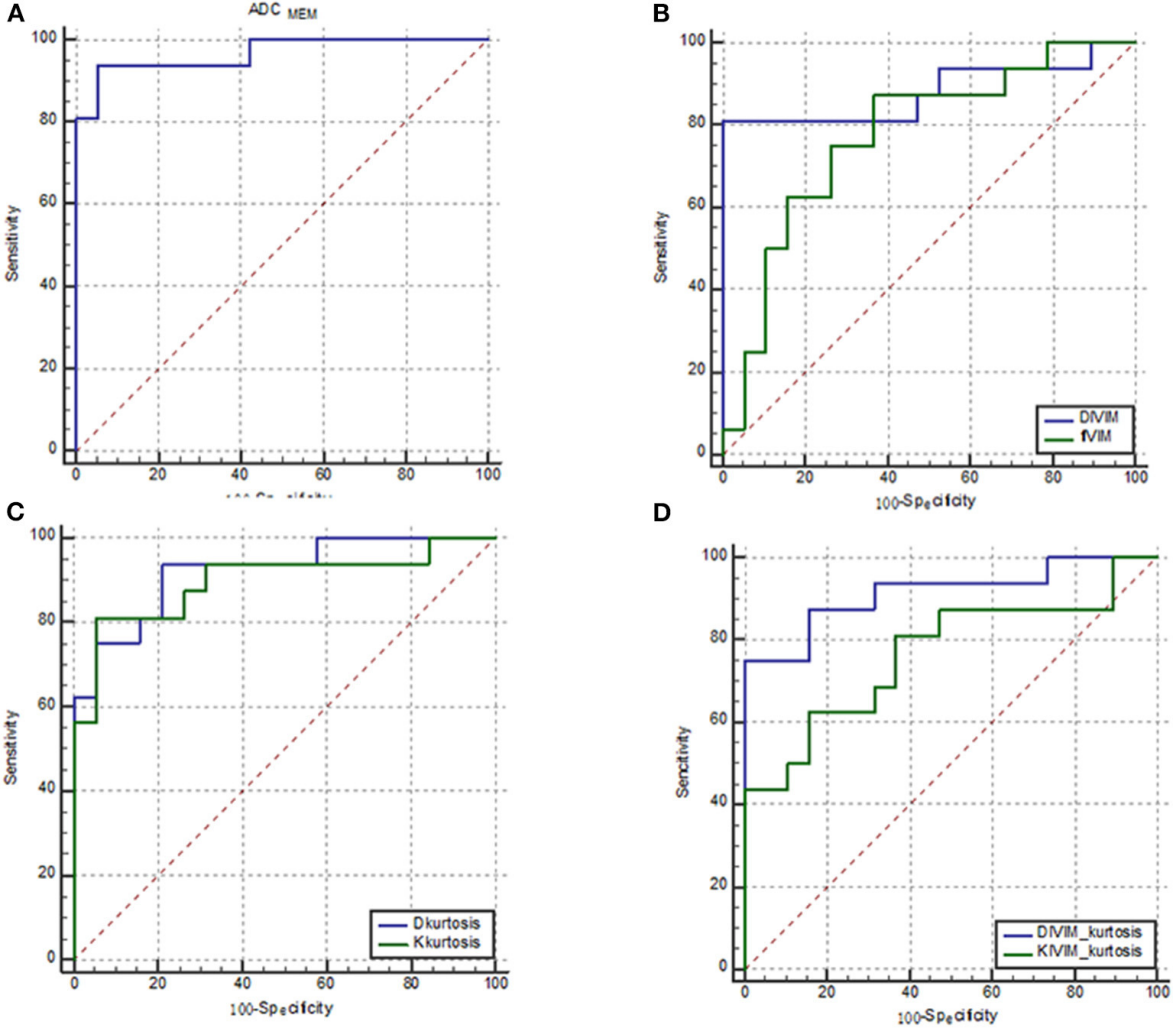
ROC curves of various parameters for identifying PCa. **(A)** ADC_MEM_; **(B)** *D*_IVIM_ and *f*_IVIM_, which were derived from the IVIM model; **(C)** *D*_kurtosis_ and *K*_kurtosis_, which were derived from the kurtosis model; and **(D)** *D*_IVIM−kurtosis_ and *K*_IVIM−kurtosis_, which were derived from the IVIM–kurtosis model. The AUC of ADC_MEM_, *D*_IVIM_, *f*_IVIM_, *D*_kurtosis_, *K*_kurtosis_, *D*_IVIM−kurtosis_, and *K*_IVIM−kurtosis_ was 0.967, 0.882, 0.773, 0.921, 0.898, 0.914, and 0.766, respectively.

The results of ROC analyses of various parameters were displayed in Figure 4. The area under the curve (AUC) of ADC_MEM_, *D*_IVIM_, *f*_IVIM_, *D*_kurtosis_, *K*_kurtosis_, *D*_IVIM−kurtosis_, and *K*_IVIM−kurtosis_ was 0.967, 0.882, 0.773, 0.921, 0.898, 0.914, and 0.766, respectively.

Table 4 shows the AUC, sensitivity, specificity, and cutoff values of the parameters. *D*_IVIM_ and *D*_IVIM−kurtosis_ exhibited relatively higher sensitivity compared with other parameters, and ADC_MEM_ showed the highest specificity among the parameters.

**Table 4:** Diagnostic performance of ADC_MEM_, *D*_IVIM_, *f*_IVIM_, *D*_kurtosis_, *K*_kurtosis_, *D*_IVIM−kurtosis_ and *K*_IVIM−kurtosis_ for differentiating of PCa from BPH and prostatitis.

**Parameter**	**AUC**	**Sensitivity (%)**	**Specificity (%)**	**Cutoff value**
ADC_MEM_ (10^−3^mm^2^/s)	0.967	94.74	93.75	0.828
*D*_IVIM_ (10^−3^mm^2^/s)	0.882	100	81.25	0.432
*f*_IVIM_ (%)	0.773	87.5	63.16	0.469
*D*_kurtosis_ (10^−3^mm^2^/s)	0.921	93.75	78.95	1.371
*K*_kurtosis_	0.898	94.74	81.25	0.836
*D*_IVIM−kurtosis_ (10^−3^mm^2^/s)	0.914	100	75	0.809
*K*_IVIM−kurtosis_	0.766	84.2	62.5	1.142

*AUC, Area under curve*.

Table 5 presents the results of ROC comparisons among the parameters. The AUC showed a significantly higher value for ADC_MEM_ than for *f*_IVIM_ and *K*_IVIM−kurtosis_ (*P* = 0.0188 and 0.0260, respectively), but the results did not reveal statistical differences among the other parameters.

**Table 5:** Results of ROC comparisons for different parameters.

	***D*_**IVIM**_**	***f*_**IVIM**_**	***D*_**kurtosis**_**	***K*_**kurtosis**_**	***D*_**IVIM-kurtosis**_**	***K*_**IVIM-kurtosis**_**
ADC_MEM_ *D*_IVIM_	0.1127	**0.0188** 0.3719	0.0581 0.5125	0.3073 0.8532	0.0801 0.5629	**0.0260** 0.2225
*f*_IVIM_			0.0682	0.2650	0.1111	0.9630
*D*_kurtosis_				0.7760	0.7010	0.1244
*K*_kurtosis_					0.8454	0.1667
*D*_IVIM−kurtosis_						0.1466

ROC, Receiver operating characteristic.

The AUC of ADC_MEM_ had a significantly higher value than that of f_IVIM_ and K_IVIM−kurtosis_. No statistically significant differences were found between the other parameters. The bold value means *p* < 0.05.

**Figure 5:**
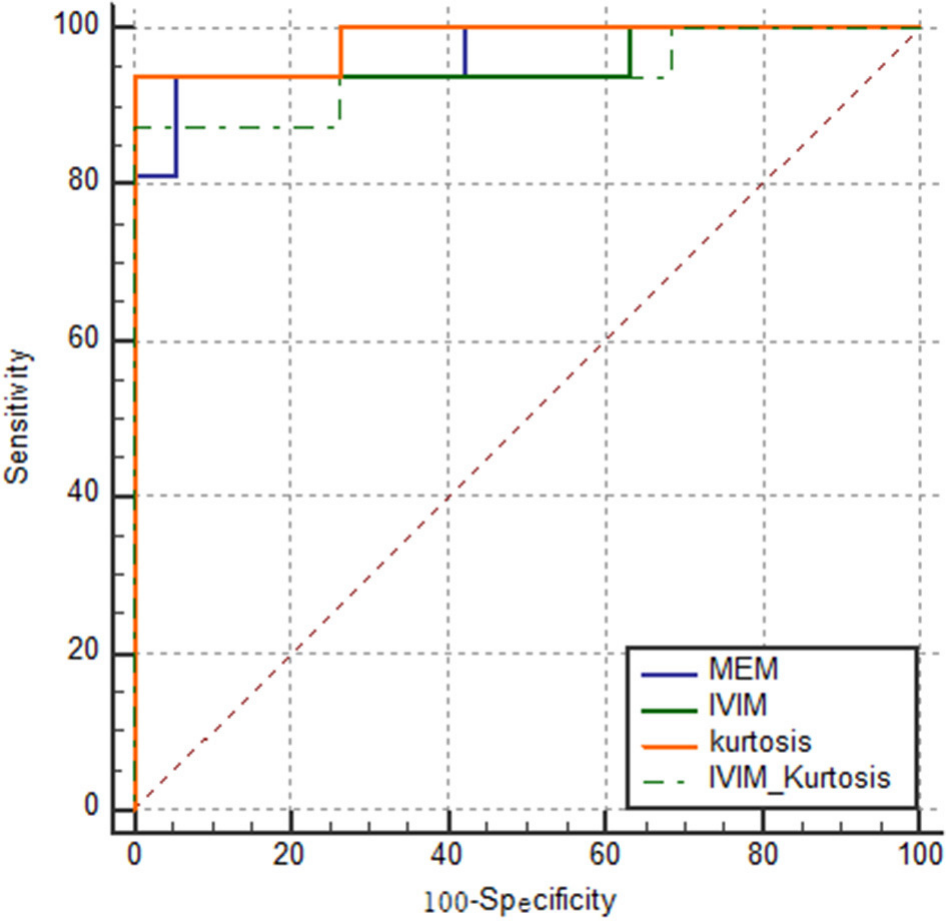
ROC curves of the four models for identifying PCa. The AUC of MEM, IVIM, kurtosis, and IVIM–kurtosis was 0.967, 0.961, 0.984, and 0.941, respectively.

The results of ROC analyses of various models are displayed in Figure 5. The AUC of the MEM, IVIM, kurtosis, and IVIM–kurtosis models was 0.967, 0.961, 0.984, and 0.941, respectively.

The results in Table 6 show the ROC comparisons among different models. The four models were found to have comparable diagnostic efficiency.

**Table 6:** Results of ROC comparisons for different models.

	**Kurtosis**	**IVIM**	**IVIM-kurtosis**
MEM	0.2670	0.7011	0.3029
Kurtosis		0.3393	0.1758
IVIM			0.2872

*ROC, Receiver operating characteristic*.

*No significant differences were found among the four models*.

Correlation of parameters with the GS are shown in Table 7; the D*_IVIM−kurtosis_ value correlated negatively with the GS (*r* = −0.649, *P* = 0.007), and *f*_IVIM−kurtosis_ and *K*_IVIM−kurtosis_ values correlated positively with the GS (*r* = 0.639, *P* = 0.008; *r* = 0.622, *P* = 0.010, respectively). The other parameters had no significant correlations with the GS.

**Table 7:** Correlation of parameters with the GS.

**MEM**	**IVIM**			**Kurtosis**		**IVIM-kurtosis**			
**ADC_**MEM**_**	***D*_**IVIM**_**	D^*^_IVIM_	***f*_**IVIM**_**	***D*_**kurtosis**_**	***K*_**kurtosis**_**	***D*_**IVIM-kurtosis**_**	D^*^_IVIM-kurtosis_	***f*_**IVIM-kurtosis**_**	***K*_**IVIM-kurtosis**_**
*P* 0.570	0.563	0.308	0.567	0.119	0.088	0.155	**0.007**	**0.008**	**0.010**
*r* −0.154	−0.156	−0.272	−0.155	−0.405	−0.440	−0.373	−0.649	0.639	0.622

*GS, Gleason score*.

Pearson test showed that the D*_IVIM−kurtosis_ value correlated negatively with the GS (r = −0.649, P = 0.007), f_IVIM−kurtosis_ and K_IVIM−kurtosis_ values correlated positively with the GS (r = 0.639, P = 0.008; r = 0.622, P = 0.010, respectively). The other parameters had no significant correlations with GS. The bold values mean p < 0.05.

**Figure 6:**
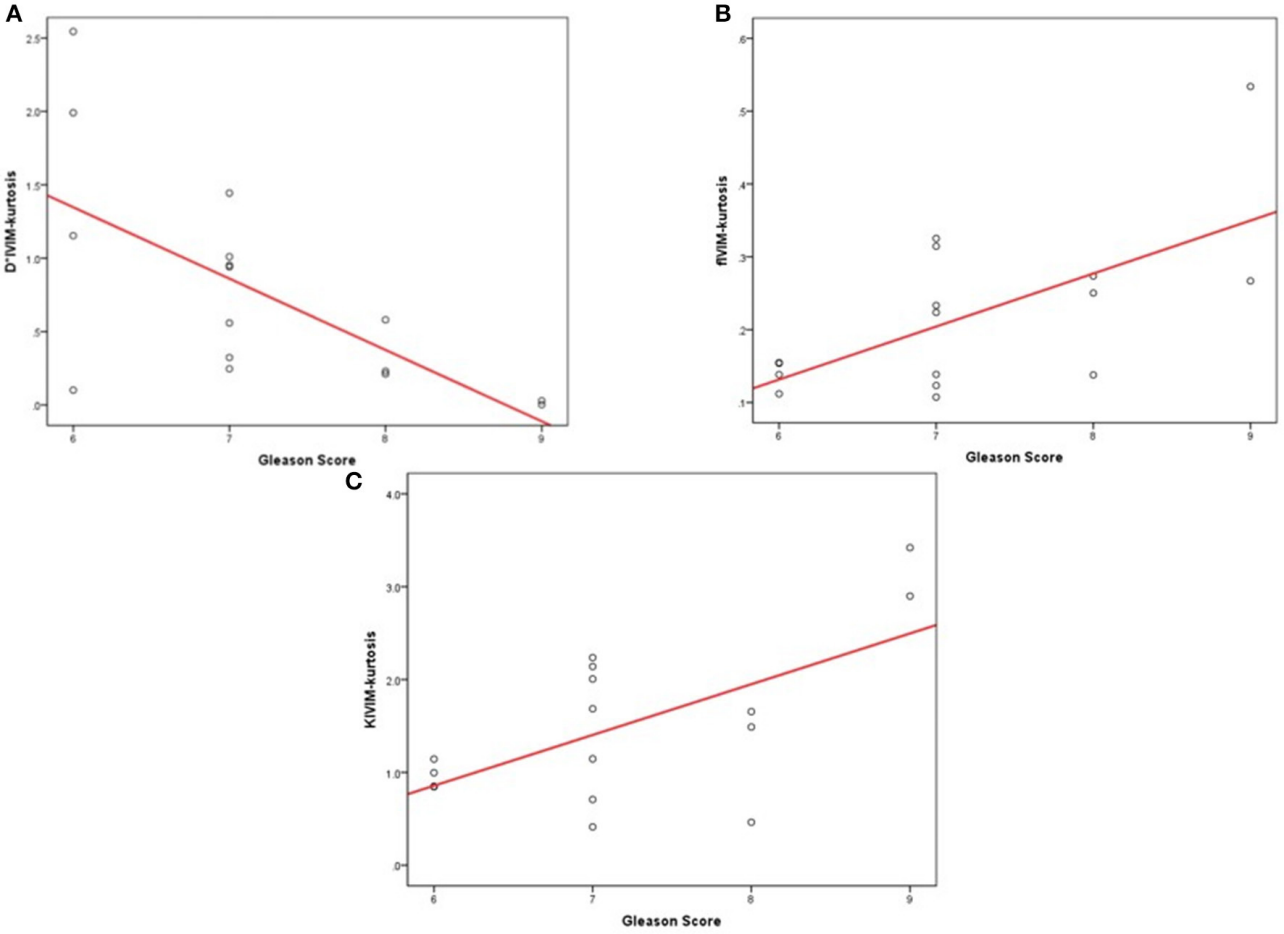
The scatter plots that showed the correlation of parameters with GS. The scatter plots that showed the correlation of parameters with GS. **(A)** D*_IVIM−kurtosis_ value correlated negatively with GS. **(B)** *f*_IVIM−kurtosis_ values correlated positively with the GS. **(C)** *K*_IVIM−kurtosis_ values correlated positively with the GS.

Figure 6 is the scatter plots that showed D*_IVIM−kurtosis_ value correlated negatively with GS, while *f*_IVIM−kurtosis_ and *K*_IVIM−kurtosis_ values correlated positively with GS.

## Discussion

The findings of this study revealed that the MEM, IVIM, kurtosis, and IVIM–kurtosis models could all be used to differentiate PCa from non-cancerous tissue.

ADC_MEM_, based on the monoexponential decay model, derives from the assumption that water molecular diffusion is a random process. The results of this study demonstrated that ADC_MEM_ was significantly lower in PCa than in non-cancerous tissue, which was in accordance with previous studies (29, 30). The lower ADC_MEM_ in PCa may be owing to increased cellularity and fibrosis.

IVIM, following a bi-exponential model, provides pure molecular diffusion parameters (*D*) and perfusion-related diffusion parameters (D^*^ and *f* ) (16). *D*_IVIM_ and *f*_IVIM_ were statistically lower in PCa compared with non-cancerous tissue, which was similar to the result of Döpfert et al. (31). However, the results of the *f* value in previous studies were various. Some studies found no significant differences among PCa, prostatitis, and BPH (16, 32); however, several studies also showed a higher *f* value in PCa (14). Such controversial results may be partially due to the poor repeatability, which may be attributed to the substantially increased heterogeneity of PCa and the intrinsically low *f* value in prostate parenchyma (19). In the present study, the D*_IVIM_ of PCa was indistinguishable from non-cancerous tissue, which corresponded to a prior report (31). The contributing factor may be that D^*^ was susceptible to measurement and noise variations. It is possible that motion across b-values inside included cases, or physiological noise may affect to the results, particularly in D^*^, to undermine its performance.

DKI, as an extension of traditional DWI, was adopted to characterize the multiexponential behavior of diffusion decay using a kurtosis-based diffusion model (33). This study showed lower *D*_kurtosis_ and higher *K*_kurtosis_ in PCa than in non-cancerous tissue, which was in accordance with previous studies (20, 23, 34). Lower *D*_kurtosis_ in PCa may be mainly because of the dense cellularity of malignant lesions. The increase in the microstructural complexity of PCa could result in increased *K* values for PCa compared with non-cancerous tissue (13).

The IVIM–kurtosis model takes into account both the IVIM and non-Gaussian diffusion effects on the diffusion-weighted signal (27), providing more information compared with the IVIM and kurtosis models. The results showed statistically lower *D*_IVIM−kurtosis_ and higher *K*_IVIM−kurtosis_ in PCa compared with non-cancerous tissues, which were similar to the results of *D*_IVIM_, *D*_kurtosis_, and *K*_kurtosis_ in differentiating PCa from non-cancerous tissue. The D*_IVIM−kurtosis_ and *f*_IVIM−kurtosis_ values had no significant differences among PCa, prostatitis, and BPH, but *f*_IVIM_ was statistically lower in PCa compared with non-cancerous tissue. The reason may be the poor measurement reproducibility of D^*^ and *f* . In this study, the results of the ROC analyses for discriminating PCa from non-cancerous tissue revealed that the AUC of ADC_MEM_ showed a higher value compared with *f*_IVIM_ and *K*_IVIM−kurtosis_, with statistical significance. However, no statistically significant differences were found between the other parameters. This may indicate that the differential diagnostic ability of ADC_MEM_ was superior to *f*_IVIM_ and *K*_IVIM−kurtosis_.

Besides the comparisons of individual parameters, the ROC was also compared among various models to find out which model has the best diagnostic accuracy.

The present study revealed that the diagnostic accuracies of the MEM, IVIM, kurtosis, and IVIM–kurtosis models were similar, indicating that the MEM, IVIM, kurtosis, and IVIM–kurtosis models all showed excellent diagnostic performances for PCa and that neither technique was superior to the other. Li et al. (32) showed that the AUC of DKI was higher than that of DWI, which was inconsistent with the results of this study. The difference may result from the different choice of multi-*b* values; therefore, it is critical to select a suitable range of multi-*b* values in studies. Unfortunately, the appropriate number and choice of *b* values are not known. Therefore, the diagnostic value of the four models with different number and range of multi-*b* values needs to be further investigated, and a large-sized sample study is necessary to confirm the diagnostic efficiency of the models. Considering the scan time and data post-processing time of IVIM, kurtosis, and IVIM-kurtosis were relatively long, so they may be not applicable to all scanners. Despite the high requirements of gradient power and software for IVIM, kurtosis, and IVIM–kurtosis models, MEM have more advantages than other models to some extent, for MEM was easily acquired in clinic with shorter scanning time, taking less resources and also being less prone to motion artifacts.

In aggressiveness assessment, the present study displayed that *f*_IVIM−kurtosis_ and *K*_IVIM−kurtosis_ values correlated positively while the D*_IVIM−kurtosis_ value correlated negatively with the GS. Meanwhile, D*_IVIM_ and *f*_IVIM_ values did not significantly correlate with the GS; the difference may result from the deviation in the evaluation of D^*^ and *f* parameters.

The *K* reflects the peaked distribution of tissue diffusivity, which increased with the complexity of the tissue's microstructure (35). The PCa tissue was filled with a destroyed glandular structure in which the cell density increases, and the intercellular space is constricted. These changes in the microstructure would all appear to represent increasing tissue complexity, leading to higher restriction to water molecule movement, which in turn results in the increased *K* value in intermediate- and high-grade PCa compared with low-grade PCa. However, the parameters derived from the MEM, IVIM, and kurtosis models had no significant correlations with the GS. The results may be attributed to the lack of samples and signal measurement errors, and the number of low-grade PCa (GS ≤ 6) was relatively small. Therefore, further studies with larger patients are needed to observe the utility of various parameters obtained from the MEM, IVIM, and kurtosis models in the aggressiveness assessment of PCa.

Moreover, the transrectal ultrasound (TRUS)-guided biopsy is accepted as the standard for the diagnosis of PCa in most previous studies. However, it has a low sensitivity (40%) (36–38), bringing out errors in the stratification of tumor and non-tumor tissues. Few studies used prostatectomy as the pathological reference, but the ROIs might not perfectly match the pathology. The use of in-bore 3-T MRI-guided biopsy in this study yielded a high PCa diagnostic rate, as it could improve the accuracy in image matching. The in-bore 3-T MRI-guided biopsy allows direct sampling of the lesions suspicious for cancer and assures that the lesion identified by MRI was the lesion evaluated by histology.

Quality of curve fitting was only conducted among three advanced diffusion models since that MEM was processed by two b values (50 and 1,500) and no curve fitting was performed. Larger AIC of Kurtosis model indicates that this model is less suitable for prostate lesions. This may suggest that perfusion caused by the rich capillary network has more contribution to the DWI signal attenuation, rather than the heterogeneous diffusion environments. Similar fitting quality between IVIM and IVIM-kurtosis model also echoes that the introduction of kurtosis component doesn't bring additional advantage into the description of signal attenuation. More histopathological analysis may be helpful for further exploration of diffusion components.

This study had some limitations. First, the patient population was relatively small, with a total of 45 ROIs, and the number of tumors with GS ≤ 6 was relatively small. Therefore, larger patient populations are needed to observe the utility of the MEM, IVIM, kurtosis, and IVIM–kurtosis models in the diagnosis and aggressiveness assessment of PCa. Second, the whole histopathology was not available in patients; this approach could allow a more precise diagnosis of PCa. However, unlike conventional TRUS-guided biopsies, this study used the in-bore MRI-guided biopsy as a reference, which improved the accuracy in biopsy location.

## Conclusion

In conclusion, the MEM, IVIM, kurtosis, and IVIM–kurtosis models were beneficial in differentiating PCa from prostatitis and BPH, but the diagnostic efficacy seemed to be similar in all four models. The IVIM–kurtosis model may be advantageous for the aggressiveness assessment of PCa compared with the MEM, IVIM, and kurtosis models.

## Data Availability Statement

The datasets presented in this article are not readily available because the policy of Beijing Hospital does not permit that. Requests to access the datasets should be directed to Chunmei Li, lichunmei4147@bjhmoh.cn.

## Ethics Statement

The studies involving human participants were reviewed and approved by Beijing Hospital ethics committee. The patients/participants provided their written informed consent to participate in this study. Written informed consent was obtained from the individual(s) for the publication of any potentially identifiable images or data included in this article.

## Author Contributions

YL, CL, KS, and MC contributed conception and design of the study. YL, XW, YC, YJ, LY, ML, WZ, JZ, and CZ organized the database. YL and CL performed the statistical analysis. YL wrote the first draft of the manuscript. CL and MC contributed to manuscript revision. All authors read and approved the submitted version.

## Conflict of Interest

KS was employed by the company Philips Healthcare. The remaining authors declare that the research was conducted in the absence of any commercial or financial relationships that could be construed as a potential conflict of interest.
